# Non-communicable diseases prevention and control by government ministries in Guyana

**DOI:** 10.3389/fpubh.2024.1369710

**Published:** 2024-09-25

**Authors:** Karen Rebecca Vanese Roberts, Carla Aparecida Arena Ventura, Alvaro Francisco Lopes de Sousa, Isabel Amélia Costa Mendes

**Affiliations:** ^1^Pan American Health Organization, Georgetown, Guyana; ^2^Ribeirão Preto College of Nursing, University of São Paulo, Ribeirão Preto, São Paulo, Brazil; ^3^Instituto de Ensino e Pesquisa, Hospital Sírio Libanês, São Paulo, Brazil; ^4^Graduate Program in Nursing, Federal University of Mato Grosso do Sul, São Paulo, Brazil

**Keywords:** disease prevention, health governance, global health, health policy, noncommunicable diseases, public policy, public health policy

## Abstract

**Background:**

Countries of the Caribbean Community signed the Declaration of Port of Spain in 2007 with the vision to stop the epidemic of non-communicable diseases (NCDs). The adoption of the Declaration by member states represented a regional effort, challenging governments, the private sector, and civil society to act together. Multisectoral actions in Guyana aimed at achieving this goal are the focus of this article, demonstrating the work of different actors in addressing the burden of NCDs in the country.

**Objective:**

To analyze multisectoral actions developed among five ministries for the implementation of the Declaration of Port of Spain 2007 in Guyana.

**Methods:**

This qualitative study was guided by the Consolidated Criteria for Reporting Qualitative Research (COREQ) checklist and conducted in five ministries (Agriculture, Education, Finance, Health, and Trade) in Georgetown, Guyana. The thematic analysis was guided by a framework consisting of four elements: context (why the policy is needed), content (what the policy is mainly about), process (how the policy was brought forward and implemented), and actors (who participates in and influences the formulation and implementation of the policy). The framework considers how these elements interconnect to shape policy development and implementation processes.

**Findings:**

Data analysis provided the researchers with insights into possible topic areas and codes for consideration during the analysis, hence a deductive approach to data analysis was used. The results highlighted the importance of coordination among government entities, national and international agencies, private actors, industry players, and civil society. Participants did not mention the use of responsibility metrics but cited mechanisms that facilitated collaboration.

**Conclusion:**

The results showed limitations in transforming multisectoral initiatives into intersectoral collaboration to achieve real integration among the different actors involved, considering the actual context and content. Actions could be more effectively implemented with significant outcomes for NCD control in Guyana.

## 1 Introduction

The right to life and the highest attainable standard of health are fundamental rights of all human beings everywhere, forming the foundation of healthy families, communities, and societies (1). Non-communicable diseases (NCDs) are the world’s leading cause of preventable morbidity and mortality, affecting both rich and poor nations alike (2). In 2019, NCDs accounted for seven out of the top 10 causes of death worldwide, a significant increase from four out of the top 10 causes in 2000. Collectively, NCDs were responsible for 74% of all deaths globally in 2019, with the world’s biggest killer being ischemic heart disease (IHD), which accounted for 16% of total deaths. Reported data indicate that, compared with advances against communicable diseases, progress in preventing and controlling premature death from NCDs has been inadequate (3).

Lower- and middle-income countries bear the greatest health burden of NCDs (4). NCDs also pose the greatest health challenges for the countries of the Caribbean Community (CARICOM), where death and disability from NCDs exceed those from infectious diseases, and the estimated premature mortality rate is twice as high compared to wealthier countries (4, 5). These small and fragile countries are unable to sustain the human and economic burden of these diseases, with people increasingly affected by NCDs during their most economically productive years—between the ages of 30 and 69 (6).

Guyana is a lower-middle-income country experiencing a growing incidence of NCDs, reflecting the global trend and accounting for over 68% of all deaths (4). Although Guyana has recently transitioned to an upper-middle-income country due to the discovery of oil, significant conditions of vulnerability persist.

Countries of the Caribbean Community (CARICOM) signed the Declaration of Port of Spain in 2007: Uniting to Stop the Epidemic of NCDs, with the aim of fostering inter-regional collaboration and facilitating multi-sector partnerships intended to serve as models for national-level partnership platforms (7). The Declaration contains 15 commitments for NCD prevention and control, addressing risk factors such as tobacco use, physical inactivity, nutrition, alcohol use, strengthening health services, surveillance, and the involvement of civil society. The content of the Declaration is summarized in Table 1. Additionally, it challenges governments, the private sector, and civil society to work together to achieve these commitments (7, 8). The adoption of the Declaration in 2007 by CARICOM member states represented a regional effort against NCDs, marking the first time member states at the regional level agreed that NCDs presented a major challenge to socioeconomic development, contributed to poverty, and threatened the achievement of health-related Millennium Development Goals (8).

**Table 1:** Areas of action in the PoSD 2007 implemented by ministry.

PoS	Areas of action	MoA	MoE	MoF	MoH	MoT
3a.	Smoke free in-door public spaces	Y	Y	Y	Y	Y
3b.	Smoke free out-door public spaces	Y	Y	Y	Y	Y
3c.	Ban on tobacco advertising and promotion	N	Y	Y	Y	Y
3d.	Ban on tobacco sponsorship	N	N	Y	Y	Y
3e.	Effective warning labels on tobacco products	N	N	Y	Y	Y
4a.	Use of revenues from tobacco for NCDs prevention programs	N	N	Y	Y	Y
5a.	Establish comprehensive plan for screening and management of NCDs	N	N	N	Y	N
5b.	Establish comprehensive plan for screening and management of NCD risk factors	N	Y	N	Y	N
5c	Facilitate access to quality care	N	N	N	Y	N
5d.	Education on prevention of NCDs	Y	Y	Y	Y	Y
6a.	Support for physical activity in schools	N	Y	N	Y	N
6b.	Implementation of physical activity at worksite	N	Y	Y	Y	N
6c.	Support for ongoing mass physical activities in communities	N	Y	N	Y	N
6d.	School feeding programs	Y	Y	Y	Y	N
6e	Promotion of healthy diet and eating	Y	Y	N	Y	N
7a.	Food and nutrition security	Y	Y	N	Y	N
7b.	Interventions to eliminate trans-fat from diet	N	N	N	Y	Y
7c.	Public education on good nutrition	Y	Y	N	Y	N
8.	Promotion of use of indigenous agricultural products	Y	N	N	Y	N
9.	Nutritional labeling	N	N	N	Y	N
10.	Increasing public spaces, parks for recreational facilities in communities.	N	Y	N	N	N
11.	Gender considerations in all programs for NCDs prevention	N	N	N	Y	N
12a.	Provision of incentives for public education programs	N	N	N	N	N
12b.	Collaboration with the media for education programs on NCDs	Y	Y	Y	Y	Y
13a.	Support for NCDs surveillance	N	N	N	Y	N
13b.	Support for NCDs research	N	Y	Y	Y	N

Y, Yes; N, No.

The World Health Organization’s Global Action Plan for the Prevention and Control of Noncommunicable Diseases (2013–2020) focuses on four major diseases (cardiovascular disease, diabetes, cancer, and chronic lung disease) and four shared risk factors—unhealthy diets, tobacco use, and the harmful use of alcohol—collectively known as the 4 × 4 framework (9). In 2019, the Global Action Plan was extended until 2030 to better align with the 2030 Agenda for Sustainable Development. In addition to the WHO Global Action Plan for the Prevention and Control of NCDs, the Strategy for NCDs Prevention and Control 2013–2019 in the Americas was developed. This regional strategy, while building on previously existing frameworks, holds the potential to substantially reduce morbidity and mortality. It places greater emphasis on raising the level of attention paid to NCDs in both the development and economic agendas of the Member States and the international community (10). It encourages a multisectoral, all-of-society approach that includes government, the private sector, academia, and civil society at regional, sub-regional, and national levels alike (3, 9).

Multisectoral action refers to actions undertaken by sectors outside the health sector, with or without collaboration with the health sector, to attain health-related outcomes or influence health determinants (11). Multisectoral partnerships are deemed necessary because the factors that influence health outcomes are complex and extend well beyond the provision of health care services. Many of these factors fall outside the authority of the Ministry of Health. As a result, accountability for the progressive realization of the right to health must be shared across the government. Coordinated action is therefore needed between ministries, different levels of government, and civil society to address complex and persistent health challenges such as NCDs (12).

Currently, it is evident that health policies are focused on achieving the Sustainable Development Goals (SDGs) by 2030. However, in the Caribbean region, despite recommendations from the Declaration of Port of Spain 2007 and the UN High-Level Meetings on NCDs that health and non-health sectors must collaborate to strengthen NCD prevention and control programs, this has not been consistently evident in many countries, including Guyana. Therefore, there is a need for information that can guide suitable interventions, as it is recognized that NCDs can only be successfully prevented and controlled through sustained linkages and partnerships with sectors outside of health (13), including trade, finance, agriculture, education, and urban planning. This study aims to analyze multisectoral collaboration among the Ministries of Agriculture, Education, Finance, Health, and Trade for the prevention and control of NCDs in Guyana since the introduction of the Declaration of Port of Spain 2007.

The aim of this study is to analyze multisectoral actions developed among five Ministries for the implementation of the Declaration of Port of Spain 2007 in Guyana.

## 2 Methods

### 2.1 Type of study

This is a qualitative study guided by the Consolidated Criteria for Reporting Qualitative Research (COREQ) checklist (14).

### 2.2 Study location

This study was conducted in five ministries (Agriculture, Education, Finance, Health, and Trade) in Georgetown, Guyana, selected due to their key portfolios within the country.

### 2.3 Study participants

The study participants were 13 senior staff members (program managers, departmental heads) from the five selected ministries.

#### 2.3.1 Inclusion criteria

Senior staff who were currently employed in one of the five selected ministries and who fulfilled decision-making roles and responsibilities related to the implementation of prevention and control interventions, policies, and programs for NCDs and risk factors within their respective ministries. The exclusion criteria were administrative, non-technical, and auxiliary staff working in the five ministries.

The authors adopted a convenience sample, and saturation was achieved with 13 individuals (15). The process of determining the sample size for this study was based on obtaining sufficient data to respond to our research problem.

### 2.4 Data collection tool

Data were collected from June to September 2021 using a semi-structured interview guide developed with consideration of the study’s goals and objectives, as well as a review of the literature on the topic. The semi-structured interview guide was piloted on five selected personnel from other agencies, including non-governmental organizations working in the prevention and control of NCDs. Feedback from the pretesting of the semi-structured interview guide indicated that the main areas of NCD prevention and control being addressed in the study needed to be clearly outlined. These inputs were integrated, and a final semi-structured interview guide was subsequently prepared with clearly outlined NCD focus areas: cardiovascular diseases, diabetes, cancer, chronic respiratory diseases, tobacco use, harmful use of alcohol, physical inactivity, and unhealthy diet.

All interviews with participants were conducted virtually due to the public health restrictions imposed because of the COVID-19 pandemic. Some participants had their interviews conducted while they were at work, while others scheduled their interviews in the evenings when they were at home.

Each interview was recorded by the interviewer with the participant’s consent. Field notes were made during and after each interview to document any special requests made by participants and to highlight any areas for follow-up by the researcher. One hour was allocated for each interview. Interview transcripts were returned to the interviewees for comments and/or corrections. All participants indicated that they were satisfied with the transcription.

The interviewer was the sole individual who coded the data. Each participant randomly selected a number from 1 to 20, which was used as a unique identifier for the participant. Numerals were used because numbers are commonly used to identify study participants in national surveys.

### 2.5 Data analysis

Thematic analysis was conducted where major and minor themes were coded, grouped, and analyzed (16). The thematic analysis for this study was guided by the HPT framework. Developed by Walt and Gilson, this simplified and rational approach helps researchers systematically understand and analyze health-related policies. The framework consists of four elements: context (why the policy is needed), content (what the policy is mainly about), process (how the policy was brought forward and implemented), and actors (who participates in and influences the formulation and implementation of the policy). It considers how these elements interconnect to shape the policy development and implementation processes (17).

During this phase, the researchers adopted a deductive approach, using pre-established categories based on the HPT framework to guide the analysis. This approach allowed for the systematic organization of the data and the clear identification of key inferences and emerging patterns.

Triangulation of themes was conducted as a means of verifying the integrity of the data, ensuring that the inferences were well-founded and accurately reflected the experiences and perceptions of the participants. Emerging patterns were reviewed and compared with existing findings in the literature, providing a solid foundation for the interpretation of the results and ensuring the validity of the study’s conclusions.

These four elements provided the researchers with insights into possible topic areas and codes for consideration during the analysis, and hence a deductive approach to data analysis was used.

### 2.6 Data processing

The data processing for this study was conducted methodically to ensure accuracy and privacy. The principal researcher collected the semi-structured interviews and then fully transcribed the recordings. Afterward, the transcriptions were thoroughly examined, and their accuracy was compared with the original recordings. To ensure data integrity, each participant was provided with their individual transcription for review, allowing for any necessary modifications and validation of their observations.

Before analysis, the data were organized into a data management system using specialized software. This software allowed for the secure entry and storage of the transcriptions, which were anonymized to protect the identities of the participants. As part of the anonymization process, each participant was assigned a unique number, enabling the coding of data without directly associating it with any particular individual. The interviews underwent a de-identification process to remove any information that could reveal the identities of the participants.

The HPT framework served as a guide during the thematic coding of the data in the analysis. Rigorous data management procedures were followed, ensuring data security from collection through to analysis. Only the researchers involved in the study had access to the data, and all precautions were taken to protect the integrity and confidentiality of the information.

### 2.7 Ethical aspects

The necessary ethical approval was obtained from the Institutional Review Board (IRB) of the Ministry of Health, Guyana, in August 2019. The protocol number is 604/2019.

## 3 Results

Thirteen participants from the five Ministries (Agriculture, Education, Finance, Health, and Trade) with 5–25 years of experience as senior staff members were recruited. More than half of the participants were from the Ministry of Health (*n* = 7). The Ministries of Agriculture and Education had two participants each, while the Ministries of Finance and Trade had 1 participant each. Most of the participants in the study were female (*n* = 9), with males being fewer in number (*n* = 4). This distribution resulted because most of the individuals who were willing to participate in the study were from the Ministry of Health and were women.

### 3.1 Demographic characteristics of the participants

Participants’ years of work experience in senior positions varied across the five ministries. In the Ministry of Health, the years of work experience of participants in senior positions ranged from 3 to 20 years. In the Ministry of Education, the years of work experience in senior positions ranged from 12 to 16 years. In the Ministry of Agriculture, the years of work experience in senior positions ranged from 10 to 20 years. In the Ministries of Finance and Trade, the study participants had worked for 5 and 10 years, respectively, within their respective ministries.

### 3.2 Content analysis

Findings were organized considering the HPT framework in four categories: Content, Context, Actors, and Policy. Content analysis of multi-sectoral actions for the implementation of the Declaration of Port of Spain 2007 showed that all participants had some knowledge of the Declaration or its elements. This knowledge was demonstrated through their statements, highlighting the importance of the Declaration as a milestone in the fight against chronic diseases in the Caribbean region. The participants emphasized that the Declaration represented a governmental commitment to making non-communicable diseases (NCDs) a priority in public policies.

This knowledge was demonstrated in the statements below:

*“*This is a Landmark Declaration about stopping chronic diseases that was signed by regional governments throughout the Caribbean to ensure policies are aligned with regard to NCDs. Governments committed to making NCDs a priority as part of policy planning highlighting issues related to health and wellness*”* (Interviewee #1, Ministry of Education).

*“*[*…*] The focus is on several prevention and control issues related to NCDs prevention, tobacco control [*…*] seen as a food and dietary issue; promoting physical activity; alcohol reduction and use, measures relating to medicines for personal living, and NCDs; information on surveillance, collecting and updating information on NCDs as often as possible" (Interviewee #3, Ministry of Health).

*“*Collaborative effort by all CARICOM countries to combat NCDs*”* (Interviewee #8, Ministry of Finance).

### 3.3 Prioritization of actions

Prioritization (i.e., priority selection, sequencing of activities, and the timing of implementation) was crucial to successful multi-sectoral collaboration. All 13 participants (100%) noted that the earmarking of priorities was overseen by an inter-ministerial government agency with private sector or industry representation, such as the NCDs Presidential Commission, or by the Cabinet of government ministers in certain instances.

There was general agreement among the interviewees that health-related issues, such as NCD prevention and treatment, were a major focus of multi-sectoral collaboration, with each ministry acting based on their unique ministerial portfolios and interests. The Ministry of Health and, to a lesser extent, the Ministry of Education were seen as central players in the scoping of most measures implemented. These measures often reflected a trade-off between public and political acceptability and effectiveness in terms of their impact on population health. However, in certain instances, the governing legislative framework of an agency was also a limiting factor, particularly affecting the Ministries of Trade and Finance.

### 3.4 Context

Situational factors are transient or “focusing events” that may influence policy (e.g., a new political party comes to power and either increases or decreases its focus on combating NCDs, or new research findings emerge on one or more NCDs). Eight participants (61%) highlighted the adverse impact of political/government changeovers on policy momentum and prioritization.

Structural factors are the relatively unchanging elements of society and may include the political system, opportunities for civil society to participate in policy discussions and decisions, the type of economy, and the employment base. Other structural factors that affect the implementation of a country’s health policy include demographic features or technological advances. The economic development status of countries affected political and public receptiveness to proposed fiscal policy solutions to NCDs. For example, the double burden of NCDs and communicable diseases experienced by Guyana was articulated by one interviewee as detrimental to the prioritization of NCDs. Additionally, all participants (100%) highlighted the harmful impact of the COVID-19 pandemic, where work on policy implementation stalled, and momentum halted as policymakers diverted their attention to the new crisis.

Cultural factors may also affect health policy. In societies where formal hierarchies are important, it may be difficult to question high officials or elder statesmen. The position of ethnic minorities or linguistic differences may lead to certain groups being poorly informed about their rights or receiving services that do not meet their particular needs.

Although many health problems are dealt with by national governments, some, like NCDs, demand cooperation between national, regional, or multilateral organizations. During the study, participants opined that international donors or agencies like the WHO and PAHO had significant and diverse influences on multi-sectoral collaboration for policy implementation processes. While international donors provided additional funds to fight NCDs, the imposition of trade agreements on the control of harmful commodities was seen by the participants as a limiting factor on consumption habits and the design of taxation measures.

“Joint planning and pooling of resources at all levels should be done with ministries and development partners such as PAHO and WHO to strategically get the desired results” (Interviewee #4, Ministry of Health).

“The Ministry of Finance, despite support from PAHO, is still unable to implement the desired system of taxation on products like tobacco and alcohol to get a grip of the situation. We have to take the trade agreements into consideration. The application of taxes ideally should deter users” (Interviewee #9, Ministry of Trade).

### 3.5 Actors

However, who is considered a policy actor, the power they wield, and how interests are negotiated in formulating policy depend on the context and process. In the analysis of actors, the following themes were generated: policy champions, the influence of industry, civil society engagement, and multilateral or international actors.

All 13 participants (100%) detailed the influence of political commitment and leadership on the design and implementation of the Declaration of Port of Spain 2007 and highlighted the importance of inter-sectoral collaboration as a necessary, though insufficient, condition for policy implementation. Several factors were cited as important supports for effective implementation: sustained endorsement and policy championing by executive levels of government, including advocacy for measures by the government and the Minister of Health, commitment to reform, the political climate, and political will. There were also instances where the scarcity of financial and human resources in the health sector resulted in the prioritization of responses to communicable diseases at the expense of actions to address NCDs.

Four of the 13 interviewees (31%) also identified that the turnover of senior health experts, who had a more sustained commitment to measures than career politicians, along with *ad-hoc* and reactive instances of policy support by politicians, led to under-resourced, conflicting, and piecemeal policy responses that undermined progress in the prevention and control of NCDs.

Industry or private sector influence was identified as a prevalent and important theme in the implementation of the Declaration of Port of Spain 2007. Spanning conceptualization, design, implementation, and sustainability of fiscal measures, industry influence encompassed a range of tactics, criticisms, and defenses utilized by local businesses whose products were subject to proposed or introduced taxation.

Four participants described industries’ use of pre-emptive action to stave off regulatory measures on products that had seen economic growth, were major causes of NCDs, and were therefore injurious to population health. Intentional framing of industry activity as a significant source of employment and contributor to economic growth was a common tactic used to influence public and political opinion on fiscal measures, particularly in the implementation of tobacco and sugary beverage control measures.

This framing was often coupled with the projection of negative ramifications for economically vulnerable primary producers, profitability, employees, and national and economic prosperity. One participant reported that such threats could potentially lead to public-private sector conflict and retaliatory trade actions by international competitors.

Nine of the 13 participants (69.2%) identified civil society engagement as integral to the successful implementation of the Declaration of Port of Spain 2007, particularly in areas related to lifestyle and behavioral changes, as well as the implementation of fiscal measures. In general, civil society groups that supported implementation included local and regional administrative bodies, government ministries, national and international research agencies, academics, special interest community groups, and non-governmental and religious organizations (NGOs) whose interests were strategically aligned. These diverse groups helped shape public and political agendas, disseminated policy-relevant information to build public awareness, support, and acceptance, and at times, countered industry claims.

A majority of the participants (11 of 13, or 85%) identified the direct support of bilateral and multilateral agencies [e.g., the Food and Agriculture Organization (FAO), World Bank, Pan American Health Organization (PAHO)], international organizations (e.g., WHO, World Bank), and other national, regional (CARICOM), and international NGOs and researchers (e.g., from universities, think tanks) as critical enablers of the implementation of the Declaration of Port of Spain 2007. The common forms of support provided by these organizations included technical cooperation and financing, linking multi-sector agencies with local counterparts, supporting multi-sectoral consensus building, and supplementing Guyana’s economic, technical, and legal capacities to overcome constraints. These forms of support were evident in the majority of NCD prevention and control measures implemented to date, especially in the design and implementation of fiscal measures related to the control of tobacco, alcohol, and beverage-related products by the Ministries of Health, Finance, Agriculture, and Trade.

### 3.6 Policy

Under the theme of inter-ministerial policy dynamics, the researchers focused on the factors that aided collaboration, the mechanisms through which collaboration was facilitated, and the barriers to collaboration among the five ministries. In examining these inter-ministerial policy dynamics, the findings revealed various mechanisms that facilitated collaboration, as well as factors that aided or hindered collaboration for the implementation of the Declaration of Port of Spain 2007.

The results identified several factors that aided collaboration among ministries. One key factor was the existence of a regional mandate to implement the Declaration at the country level. As a result, all government ministries felt a sense of obligation to collaborate in order to fulfill the country’s reporting requirements, as illustrated in the quotes outlined below:

“Being knowledgeable and conscious of the country's mandate and the overlap with other ministries […], in the nation’s interest-for the good of the country” (Interviewee #5, Ministry of Health).

**Figure 1:**
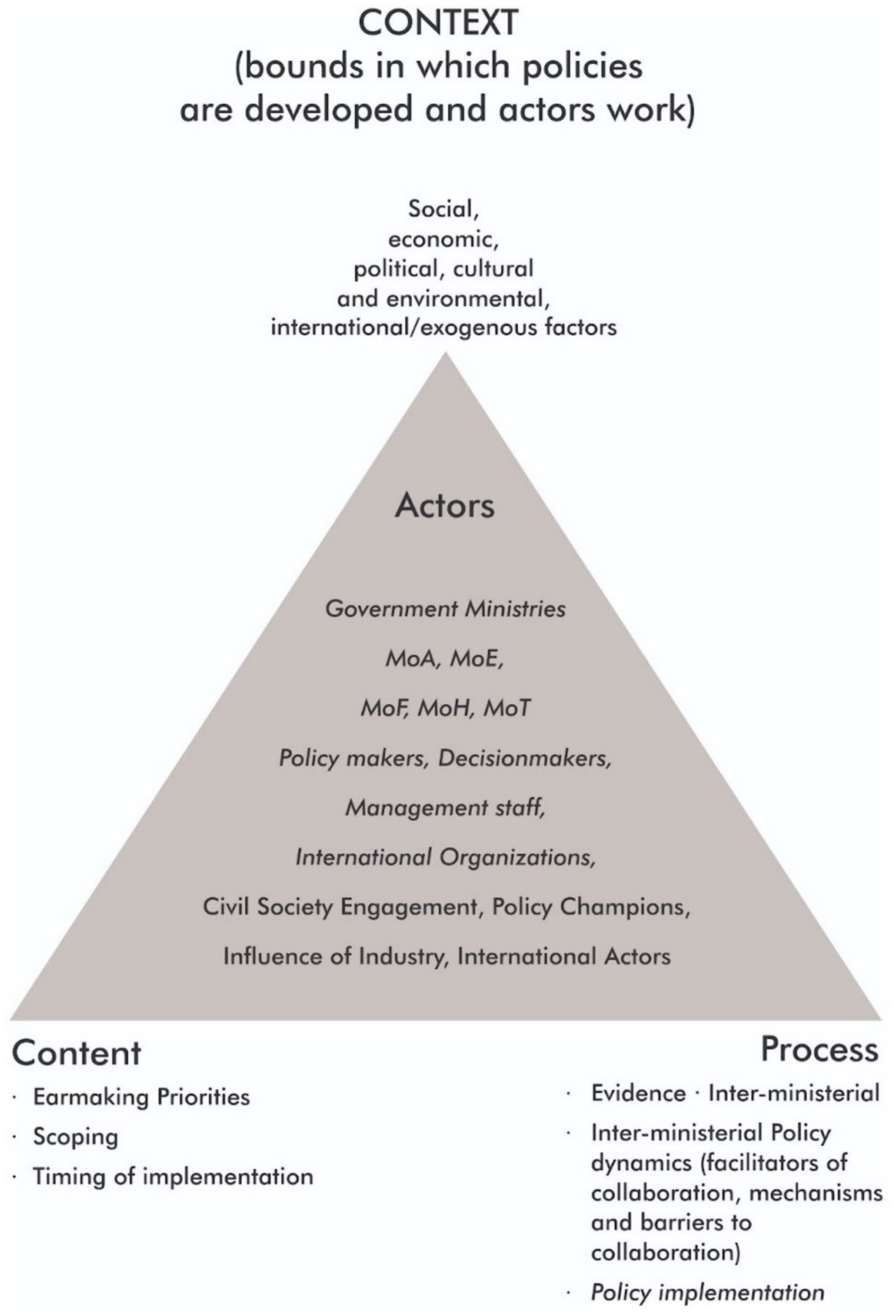
Analysis of collaboration for the implementation of the Declaration of Port of Spain 2007—Adjusted HPT Policy Triangle Framework by Walt and Gilson (17).

Figure 1 shows the elements of the Health Policy Triangle framework, a description of each of the elements and the identified themes that resulted from the thematic analysis of data gathered during the conduct of this study.

## 4 Discussion

Multisectoral actions regarding the implementation of the Port of Spain Declaration in Guyana were the focus of this article, highlighting the efforts of various actors in addressing the burden of NCDs in the country. The results demonstrated the importance of coordination among government bodies, national and international agencies, as well as private actors such as industry players and civil society.

In this sense, it is worth mentioning other similar initiatives that focus on the significance of multisectoral actions in the global response to the increase in NCDs, emphasizing the need for cooperation between various government and private sectors to overcome challenges that go beyond the control of the health sector. Three case studies were the focus of a multinational study (18): tobacco legislation in Nigeria, salt reduction in the United Kingdom, and the regulation of edible oils in Iran. The results show that, in addition to highlighting the need for strong political leadership and early consultative engagement with stakeholders, the reconciliation of conflicting values and priorities is crucial for the success of these initiatives.

In another study conducted in Tanzania (19), the government demonstrated a strong commitment by developing specific NCD units within the Ministry of Health and implementing national strategic plans and research agendas. The growing burden of NCDs in low- and middle-income countries poses similar challenges for both Guyana and Tanzania. According to both studies, other nations should adopt local strategies that align available resources with innovative and coordinated initiatives to address NCDs sustainably. Tanzania also established specific NCD units within the Ministry of Health to coordinate and strengthen these activities, while Guyana focuses on implementing the Declaration of Port of Spain 2007 through collaboration between ministries such as Health, Trade, Agriculture, Finance, and Education. In both scenarios, centralized coordination and stakeholder engagement are viewed as essential components for the success of NCD policies.

The application of the multisectoral approach (MSA) for the control of NCDs in the state of Uttar Pradesh, India (20), revealed that although the relevance of NCDs is recognized, there is a lack of full awareness of MSA guidelines, and health considerations are often not included when formulating sectoral policies. However, these sectors expressed their willingness to work together on NCD prevention initiatives. The health sector, however, understands the fundamentals of MSA but shows a lack of proactivity in encouraging its implementation. A common platform is essential to coordinate actions among the sectors, with active leadership and participation from all stakeholders. The lack of clarity and specific resources in both health and non-health sectors has hindered the implementation of MSA so far.

The lessons learned from the control of NCDs by government ministries in Guyana are relevant to our study on this prevention. Both studies highlight the challenges of implementing a multidisciplinary approach to address NCDs. The study in India highlights the lack of awareness and clarity among non-health sectors regarding their responsibilities in MSA, while the study in Guyana emphasizes the importance of coordination between ministries such as Health, Trade, Agriculture, Finance, and Education. The lack of a specific platform for coordinating multisectoral activities is a significant barrier in both contexts. In addition, greater leadership and proactive engagement from the health sector are needed in both Guyana and India to integrate actions across several sectors and ensure an effective response to NCDs.

Studies analyze metrics of responsibility for the implementation of policies, aiming to contribute to a more comprehensive understanding among governments and societies to achieve established goals, thereby generating discussions at local, regional, and global levels (21–24).

In this study, participants did not mention specific metrics, but they cited mechanisms that facilitated collaboration, such as the national mandate and commitment, which must be adhered to by each country in the region for the implementation of the Declaration’s targets. Participants emphasized a sense of obligation to collaborate in order to fulfill country reporting requirements.

Given that NCDs are the leading global factors for morbidity and mortality, countries have the responsibility to implement policies that effectively address these challenges. Despite contrasting political and financial interests (25), there is a crucial need for political balance to ensure access to health as a fundamental right, which health systems require.

In this context of weak democratic governance, the role of civil society is pivotal in the review of policies and the implementation of actions related to tobacco and alcohol control, national plans and policies regarding NCDs, and access to medication.

To empower civil society to take a leading role, it is essential to influence the context (situational, structural, and cultural factors), promoting investments in education and strategic community programs to foster knowledge and attitudes, empowering communities to exercise their rights and assume co-responsibility in supervising the implementation of local policies for NCD control. Community engagement is a powerful mechanism for building social networks that promote social control initiatives related to health policies, such as the control of NCDs (26).

Regarding context, it is important to note that situational factors are more easily influenced. The results showed that some structural and cultural factors require time and political will, with changes being incrementally implemented. Therefore, formal hierarchies and the position of ethnic minorities must be considered in the design and implementation of policies and educational programs for NCDs.

In multisectoral collaboration, the content of the policies and programs must consider the above-mentioned factors. The involvement of the Ministries of Health, Trade, Agriculture, Finance, and Education in the implementation of the Port of Spain Declaration in Guyana motivated the design of important collaboration strategies focusing on health-related issues. In this process, each ministry acted based on its unique portfolio and interests toward common goals. The results demonstrated that the selection of priorities, sequencing of activities, and timing of implementation were extremely relevant to their collaboration. Additionally, the results showed limitations in the process of transforming multisectoral initiatives into intersectoral collaboration, for the achievement of real integration among the different actors involved, considering the actual context and content. Through intersectoral collaboration, policies could be more deeply discussed and negotiated among the different actors and sectors of society, particularly given the diversity of Guyanese populations. This could lead to more effective implementation and significant outcomes in NCD control in the country.

Furthermore, a coalition of partnerships needs to be clearly envisioned and included in the government’s action plan, with intensified efforts, as its success paves the way for achieving other relevant SDG targets (27–29).

This study has limitations, primarily focused on the fact that it only considered government bodies (ministries) and did not include other important actors in multisectoral collaborations, such as the private sector and civil society.

## 5 Conclusion

This study shows that there has been collaboration among five ministries in Guyana (Agriculture, Education, Finance, Health, and Trade) for the implementation of select areas of action in the Declaration of Port of Spain 2007. There were some areas of the Declaration where only a few ministries were collaborating for implementation, as well as areas where no collaboration was taking place, with only the Ministry of Health involved in implementation.

Key multisectoral interventions, policies, and joint programs undertaken among the Ministries of Agriculture, Education, Finance, Health, and Trade for the implementation of the Declaration of Port of Spain 2007 in Guyana include tobacco control measures (e.g., smoke-free indoor and outdoor public spaces; bans on tobacco advertising, sponsorship, and promotion; adoption of warning labels; and directing revenues from the sale of tobacco products to fund NCD prevention programs) to address lifestyle and behavioral changes. Other joint programs included education, awareness, and prevention initiatives, such as support for physical activity in schools, collaboration with the media for educational programs, establishment of school feeding programs, and the promotion of healthy diets.

Various mechanisms, including commissions, councils, committees, and technical working groups, were used to facilitate collaboration among the five ministries. A strong commitment to the regional mandate to implement the Declaration at the country level was a major facilitator for collaboration, as all government ministries felt a sense of obligation to collaborate in order to fulfill country reporting requirements.

## Data Availability

The raw data supporting the conclusions of this article will be made available by the authors, without undue reservation.
